# Immortalized Adult Rodent Schwann Cells as In Vitro Models to Study Diabetic Neuropathy

**DOI:** 10.1155/2011/374943

**Published:** 2011-06-13

**Authors:** Kazunori Sango, Hiroko Yanagisawa, Shizuka Takaku, Emiko Kawakami, Kazuhiko Watabe

**Affiliations:** Department of Sensory and Motor Systems (ALS/Neuropathy Project), Tokyo Metropolitan Institute of Medical Science, Setagaya-ku, Tokyo 156-8506, Japan

## Abstract

We have established spontaneously immortalized Schwann cell lines from normal adult mice and rats and murine disease models. One of the normal mouse cell lines, IMS32, possesses some biological properties of mature Schwann cells and high proliferative activities. The IMS32 cells under hyperglycemic and/or hyperlipidemic conditions have been utilized to investigate the pathogenesis of diabetic neuropathy, especially the polyol pathway hyperactivity, glycation, increased oxidative stress, and reduced synthesis of neurotrophic factors. In addition to the mouse cell lines, our current study focuses on the characterization of a normal rat cell line, IFRS1, under normal and high glucose conditions. These Schwann cell lines can be valuable tools for exploring the detailed mechanisms leading to diabetic neuropathy and novel therapeutic approaches against that condition.

## 1. Introduction

 During the development and regeneration of the peripheral nervous system (PNS), Schwann cells are responsible for providing trophic support for the growth and maintenance of neurons and ensheathing their axons in either a myelinating or an unmyelinating form [1]. Schwann cell abnormalities as a result of hyperglycemia can be a cause of nerve dysfunction, such as reduced nerve conduction velocity, axonal atrophy, and impaired axonal regeneration [2, 3]. In addition to the hyperglycemia-related mechanisms of nerve injury, recent experimental and clinical studies suggest that dyslipidemia due to obesity and type 2 diabetes can play a role in the development and progression of peripheral neuropathy [4, 5]. Although the detailed pathogenesis remains unclear, metabolic alterations in Schwann cells under hyperglycemic and/or hyperlipidemic conditions appear to be, at least in part, associated with polyol pathway hyperactivity, glycation of cellular proteins and lipids, increased oxidative stress, altered protein kinase C activity, and reduced supply of neurotrophic factors [3–8]. Moreover, impaired lipid and cholesterol metabolism in Schwann cells under diabetic conditions may affect the structure and function of peripheral myelins [9, 10].

 Cultured Schwann cells can be valuable tools for exploring the pathogenesis of diabetic neuropathy and the strategy for its prevention and treatment. Since some biological properties of Schwann cells change with maturation and aging [11, 12], culture systems of mature Schwann cells appear to mimic diabetic neuropathy and other neurodegenerative diseases better than those of immature cells. Compared with a considerable number of articles on Schwann cells from embryonic and neonatal animals [13, 14]; however, far fewer studies have been made with primary cultures of Schwann cells from adult animals [15, 16]. One of the reasons for this seems to be problems in isolation of Schwann cells from mature peripheral nerves; fully developed epineurium and perineurium with substantial amounts of connective tissue make it difficult and time consuming to get good yields of Schwann cells and sufficiently eliminate fibroblasts from the culture. To avoid such a long process of the primary culture, many investigators have established cell lines from Schwannoma cells (e.g., JS1 [17, 18]) and long-term cultured Schwann cells by transfection of oncogenes such as SV40 large T antigen (e.g., MS1 [19]) or by spontaneous immortalization [20–22]. These lined cells rapidly proliferate to increase the cell number and are more suitable for molecular and biochemical analyses than primary cultured Schwann cells. On the other hand, the degree of differentiation and phenotypic expression of these Schwann cell lines differ from each other; continuous cell lines that possess distinct phenotypes of mature Schwann cells are desirable for the study of diabetic neuropathy. In this paper, we summarize the biological features of these cell lines, especially IMS32 from adult ICR mice [21] and IFRS1 from adult Fischer 344 rats [22], and discuss their usefulness as in vitro models of diabetic neuropathy. IMS32 cells seem to be one of the best-characterized Schwann cell lines at present and have advantages for the study of polyol pathway-related abnormalities under diabetic conditions [23]. Although we have limited information about IFRS1 cells at present, their fundamental ability to myelinate axons in coculture with adult dorsal root ganglion (DRG) neurons [22] will be beneficial for the study of neuron-Schwann cell interactions under normal and diabetic conditions.

## 2. Immortalized Adult Mouse Schwann Cells IMS32

### 2.1. Establishment of Immortalized Schwann Cells

 In the primary cultures of DRG and peripheral nerves derived from adult ICR mouse, their morphology and immunocytochemical staining identified neurons, Schwann cells, and fibroblasts. These neurons were viable for at least 3 weeks in vitro. Following repeated treatment of primary cultures with antibody to mouse Thy-1.2 and rabbit complement for the first 2-3 weeks in vitro, the cultures consisted of >95% Schwann cells and <5% fibroblasts. These cells were fed twice a week and passaged once in 2 weeks. After 6–8 months in vitro, spontaneously developed colonies were observed. They were separated using cloning rings, and five different cell lines (IMS8, 13, 26, 29, and 32) were obtained. One of the cell lines, IMS32, was further characterized [21]. Although the detailed mechanism of spontaneous immortalization of these Schwann cells is still unknown, it has been reported that rat Schwann cells can divide indefinitely under the appropriate culture conditions [24]. So far, we have been able to obtain immortalized Schwann cell lines from mice of ICR [19, 21], BALB/c [25], and C57BL strains [26–29] (Table 1)and rats of Fischer 344 [22], Wistar, and Sprague-Dawley strains (Watabe et al., unpublished data). Taking these findings into consideration, it is likely that the spontaneous immortalization of long-term cultured Schwann cells is a general phenomenon in mice and rats, regardless of their strains.

### 2.2. Biological Features of IMS32

**Figure 1:**
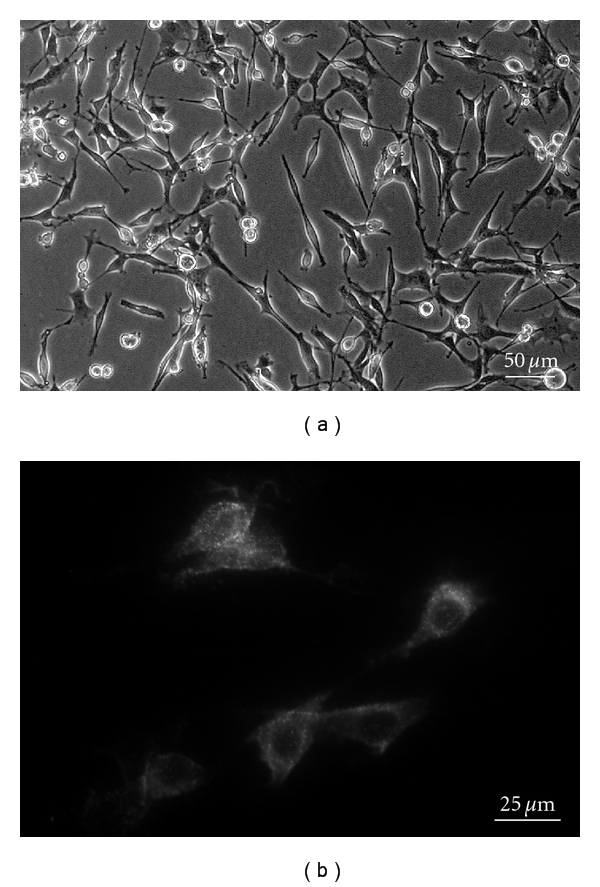
IMS32 showed distinct Schwann cell phenotypes such as spindle-shaped morphology (a) and immunoreactivity to p75 ^NTR^ (b).

 IMS32 cells showed distinct Schwann cell phenotypes, such as the spindle-shaped morphology (Figure 1(a)) and the expression of glial cell markers (e.g., S100, glial fibrillary acidic protein (GFAP) (not shown), and p75 low-affinity neurotrophin receptor (p75 ^NTR^) (Figure 1(b)), transcription factors crucial for Schwann cell development and peripheral myelin formation (e.g., PAX3, Krox20, Oct6, and Sox10), and neurotrophic factors required for the survival of neurons and the maintenance of neuron-Schwann cell interactions (e.g., nerve growth factor (NGF), brain-derived neurotrophic factor (BDNF), glial cell line-derived neurotrophic factor (GDNF), and ciliary neurotrophic factor (CNTF)) [21, 25]. Similar to primary and long-term cultured Schwann cells, IMS32 cells exhibit mitogenic responses to several growth factors (e.g., platelet-derived growth factor BB homodimer (PDGF-BB), acidic and basic fibroblast growth factor (aFGF and bFGF), and transforming growth factor (TGF)-*β*1, 2 [21]. In contrast, IMS32 cells are different from primary and long-term cultured Schwann cells in that the former were not contact inhibited and formed ball-shaped subcolonies when cultures reached confluence [21, 30]. We failed to show that the cell line could myelinate a mouse axon, in the same way as endogenous Schwann cells in the peripheral nerves and primary cultured Schwann cells (data not shown). We speculate that the high proliferative activity of IMS32 cells might impede continuous and stable neuron-Schwann cell interactions, which usually take 4 weeks or longer to form myelin sheath. In spite of those differences from normal Schwann cells, IMS32 cells have been utilized to investigate the action mechanisms of various molecules involved in peripheral nerve regeneration (e.g., CNTF [31, 32], sonic hedgehog [33], and galectin-1 [30]) and the pathogenesis of diabetic neuropathy, as described below.

### 2.3. IMS32 as a Valuable Tool for the Study of Diabetic Neuropathy

#### 2.3.1. Polyol Pathway

**Figure 2:**
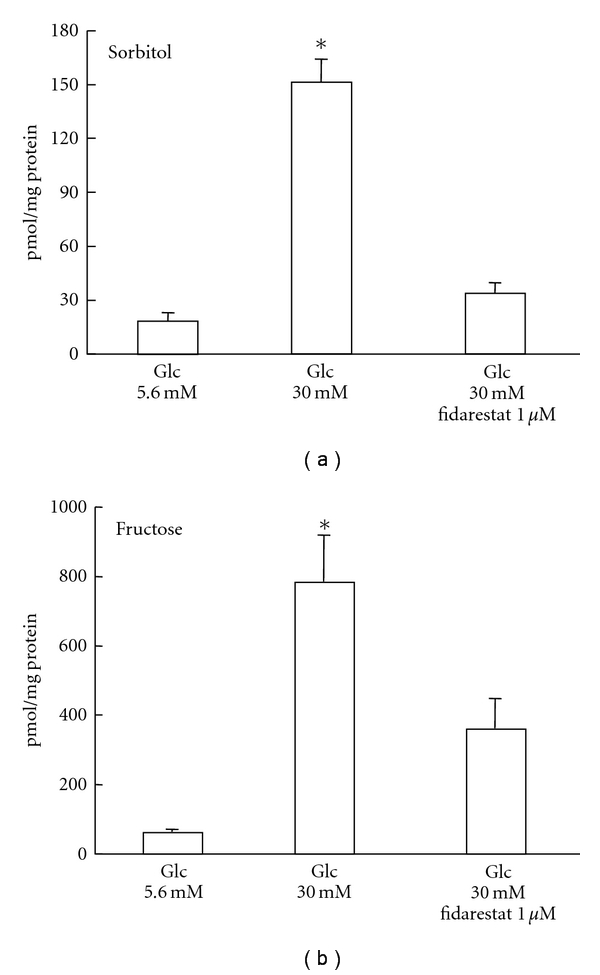
Intracellular contents of sorbitol (a) and fructose (b) in IMS32 cells after 7 days of exposure to normal (Glc 5.6 mM) and high glucose conditions (Glc 30 mM) in the presence or absence of an AR inhibitor, fidarestat (1 *μ*M). Values represent the mean + SEM of 6 experiments. *P < .01 as compared with [Glc-5.6 mM] or [Glc-30 mM/fidarestat 1 *μ*M] (modified from [21]).

The role of Schwann cells in diabetic neuropathy is often discussed in relation to the polyol pathway hyperactivity. Aldose reductase (AR), the first enzyme in the polyol pathway, is localized to Schwann cells in the peripheral nerves [34] and the increased glucose flux into the pathway via AR and the subsequent accumulation of sorbitol in Schwann cells can directly or indirectly affect nerve functions [3, 6]. A cell line from rat Schwannoma, JS1 [17], and primary cultured adult rat Schwann cells [16] have been introduced to study polyol metabolism; however, these cells did not display intracellular sorbitol accumulation or enhanced AR expression under high glucose (25–30 mM) conditions unless hyperosmotic stress (greater than 100 mM) was applied. In contrast to those studies, we observed increased AR mRNA/protein expression and marked accumulation of sorbitol and fructose in IMS32 cells cultured under a high glucose (30 mM) condition. Further, application of an AR inhibitor, fidarestat (Sanwa Kagaku Kenkyusho, Nagoya, Japan), to the high glucose medium diminished the intracellular sorbitol content to a level close to a normal (5.6 mM) glucose medium [23, 35] (Figure 2). These findings led us to believe that the culture of IMS32 under high glucose conditions is a suitable *in vitro* model for the study of polyol pathway-related abnormalities in diabetes. It remains to be elucidated why an increase in the glucose concentration to 20–30 mM, corresponding to the plasma level in poorly controlled diabetic patients, accelerated the polyol pathway in IMS32 [23, 35] but not in other cultured Schwann cells [16, 17]. Sorbitol is converted to fructose by sorbitol dehydrogenase (SDH), the second enzyme in the polyol pathway. The mRNA expression of SDH in the primary cultured Schwann cells was much lower than that of AR and was not upregulated by exposure to a 30 mM glucose condition [36]. In addition, treatment with an SDH inhibitor, SDI-158, had no effect on the intracellular sorbitol levels [16]. On the other hand, the extracellular sorbitol level in that condition was increased significantly compared with that in a 5.6 mM glucose condition [16]. These findings suggest that sorbitol is released from the cells into the media by an unidentified transport mechanism under normal and high glucose environments. It is of interest to note that treatment with SDH inhibitors augmented accumulation of sorbitol in the primary cultured Schwann cells and JS1 cells exposed to hyperosmotic conditions [16, 18]; SDH, as well as AR, might function as a protective molecule against osmotic stress. In contrast to those cells, the upregulation of the mRNA expression for AR and SDH, together with conspicuous increases of intracellular sorbitol and fructose levels, was observed in IMS32 under a 30 mM glucose condition [23]. It seems possible that IMS32 cells possess a much higher capacity than other Schwann cells to store sorbitol and other glucose-derived metabolites. It is also noteworthy that immortalized Schwann cells were established from not only normal adult mice, but also from mouse models of lysosomal storage diseases such as Niemann-Pick disease type C (NPC) [25, 26], globoid cell leukodystrophy (Twitcher) [27], G_M2_ gangliosidosis (Sandhoff disease) [28], and Fabry disease [29]. The cells originated from those mouse models were able to survive and proliferate in culture despite the progressive accumulation of undergraded substrates in the cytoplasm.

#### 2.3.2. Glycation

 In addition to the polyol pathway hyperactivity, nonenzymatic glycation of structural and functional proteins in the peripheral nervous system is another important cause of diabetic neuropathy. Research suggests that advanced glycation end products- (AGEs-) induced modification of myelin proteins, cytoskeletal proteins, and extracellular matrix proteins contributes to segmental demyelination, axonal degeneration, and impaired axoplasmic transport and regenerative activity [37]. The receptor for AGE (RAGE) is localized to axons and Schwann cells in the peripheral nervous system [38], and AGE-RAGE interactions under diabetic conditions are involved in Schwann cell dysfunction through activating several signaling pathways (e.g., NF-*κ*B and PKC*β*II pathways) [37–39]. RAGE is also known to have multiple ligands besides AGEs (e.g., serum amyloid A (SAA), S100/calgranulins, and high mobility group box-1 (HMGB1)) [40], and their interactions may alter the functional properties of Schwann cells [41, 42]. Our DNA microarray and RT-PCR analyses revealed high glucose-induced upregulation of SAA3 mRNA in IMS32 cells [23], but its relevance to glycation or other neurodegenerative changes remains unknown.

 It is also important to note that some AGE precursors, such as methylglyoxal (MG) and 3-deoxyglucosone (3DG), are generated via the polyol pathway [43]. Thus, enhanced glucose flux into the polyol pathway can be a cause of excess formation of MG and 3DG, which in turn accelerates the formation of AGEs and reactive oxygen species (ROS). These compounds, when applied exogenously, exhibited potent cytotoxicity to primary cultured neurons [44] and Schwann cells [45], and IMS32 cells [35, 46]. MG has been shown to activate caspase-3 and c-Jun-N-terminal kinase (JNK) and enhanced intracellular ROS formation in IMS32 cells [46].

#### 2.3.3. Oxidative Stress

 We observed the upregulation of the oxidative stress markers, such as 4-hydroxy-2-nonenal (4HNE), acrolein (ACR), and hexanoyl lysine (HEL), in IMS32 under high glucose conditions [35]. 4HNE, one of the 4-hydroxyalkenals generated from hydroxyperoxides, can bind to histidine, lysine, and cysteine residues of various proteins, thereby altering the protein structure and function. In a recent study by Akude et al. [47], 4HNE leads to the modification of key mitochondrial proteins through adduct formation and impairs mitochondrial function and axonal outgrowth in adult DRG neurons. ACR is more reactive than 4HNE and has exerted direct neurotoxic activities on cultured hippocampal neurons, affecting mitochondria [48]. The induction of ACR in the retina [49] and kidney [50] under diabetic state suggests its involvement in the development and/or progression of diabetic complications. HEL, a novel lipid hydroperoxide-modified lysine residue, is thought to be a useful biomarker for the initial stage of lipid peroxidation [51]. The urinary HEL from patients with diabetes was significantly higher than that from nondiabetics [52]. In addition, the HEL levels in the vitreous fluid and serum were significantly higher in patients with proliferative diabetic retinopathy than those in nondiabetic patients (Macular hole) [53]. These findings suggest the involvement of 4HNE, ACR, and HEL in the pathogenesis of diabetic neuropathy and/or other complications. In our study, exposure to high glucose environments can accelerate the reactions of lipid peroxidation and the production of the oxidative stress markers in Schwann cells [35]. Oxidative damage induced by mitochondrial dysfunction under hyperglycemic conditions may trigger the apoptotic cascade [54]; however, it is controversial whether hyperglycemia is a potent inducer of apoptosis in cultured Schwann cells [55, 56]. We failed to observe high glucose- (30 mM and 56 mM) induced apoptosis of IMS32. These findings, together with those in a previous study [56], suggest that the load of high glucose on Schwann cells does not appear to be a sufficient inducer of cell death unless subjected to further insults [57]. It is noteworthy that ischemia-reperfusion injury to peripheral nerves of STZ-diabetic rats caused a marked increase in apoptotic Schwann cells [58]. A recent study by Padilla et al. [59] indicate that palmitic acid- (PA-) induced lipotoxicity accelerates the apoptotic cascade in immortalized adult rat Schwann cells [20] under normal and high glucose conditions; the lipotoxic effect is more prominent in the culture under high glucose conditions. Consistent with that study, Suzuki et al. [60] observed the PA-induced apoptosis in primary cultured Schwann cells and IMS32 cells under normal glucose (5.5 mM) conditions. According to those reports, ER stress combined with oxidative stress might be involved in the lipotoxicity in Schwann cells.

#### 2.3.4. Synthesis of Neurotrophic Factors

 NGF and the related members of neurotrophin (NT) family (BDNF, NT-3, and NT-4/5) are produced in target tissues of the peripheral nervous system and transported retrogradely to the neuronal perikarya, where they exert their actions [61]. Reduced supply of NGF to the sensory and sympathetic neurons can be a cause of small sensory and autonomic fiber dysfunction, whereas deficiency of NT-3 appears to be involved in large fiber dysfunction in diabetic neuropathy [62]. In addition, deficient supply of these molecules under diabetic conditions is associated with impaired axonal regeneration after injury [63]. A few studies suggested reduced synthesis and secretion of these molecules in primary cultured Schwann cells under diabetic conditions [64, 65], but its precise mechanisms remain unclear.

 According to a recent study by Tosaki et al. [66], conditioned medium (CM) obtained from IMS32 under the high glucose condition (30 mM) showed lower NGF concentration and neurite-outgrowth activity for cultured adult mouse DRG neurons than that under the normal glucose condition (5.5 mM). These findings imply that the reduced NGF synthesis by Schwann cells under hyperglycemic conditions can be a cause of impaired axonal regeneration and dysfunction of small-fiber sensory and autonomic fibers [67]. However, our DNA microarray analysis failed to show significant downregulation of mRNA expression for NGF and other neurotrophic factors in IMS32 under high glucose conditions [23]. In addition, our previous study employing PC12 cells revealed that the neurite outgrowth activity of NGF, but not CM from IMS32, was attenuated by cotreatment with anti-NGF neutralizing antibody [21]. These findings suggest the combined effects of multiple neurotrophic factors, other than NGF, in case of CM. The quality and quantity of neurotrophic molecules secreted from IMS32 cells under hyperglycemic and/or hyperlipidemic conditions will need to be evaluated more precisely in the future study.

## 3. Immortalized Schwann Cells from Murine Disease Models

 Establishment of the Schwann cell lines from murine disease models may greatly facilitate the studies of the cellular mechanisms of their PNS lesions in the relevant diseases. Besides Schwann cell lines derived from normal (wild-type) mice, we have established spontaneously immortalized mouse Schwann cell lines from murine models of NPC [25, 26], Krabbe disease [27], Charcot-Marie-Tooth disease [25], neurofibromatosis [25], Sandhoff disease [28], and Fabry disease [29] (Table 1). These cell lines retain genomic and biochemical abnormalities, sufficiently representing the pathological features of the mutant mice. In a similar manner, we plan to establish immortalized Schwann cells from AR-deficient mice [68], RAGE-deficient mice [69], and the murine models of type 2 diabetes (e.g., db/db [70] and ob/ob [71]). In addition to IMS32 cells, those cell lines would provide useful information about the respective pathogenetic mechanisms and their crosstalks leading to diabetic neuropathy and the novel therapeutic approaches against that condition.

## 4. Immortalized Adult Rat Schwann Cells IFRS1

**Figure 3:**
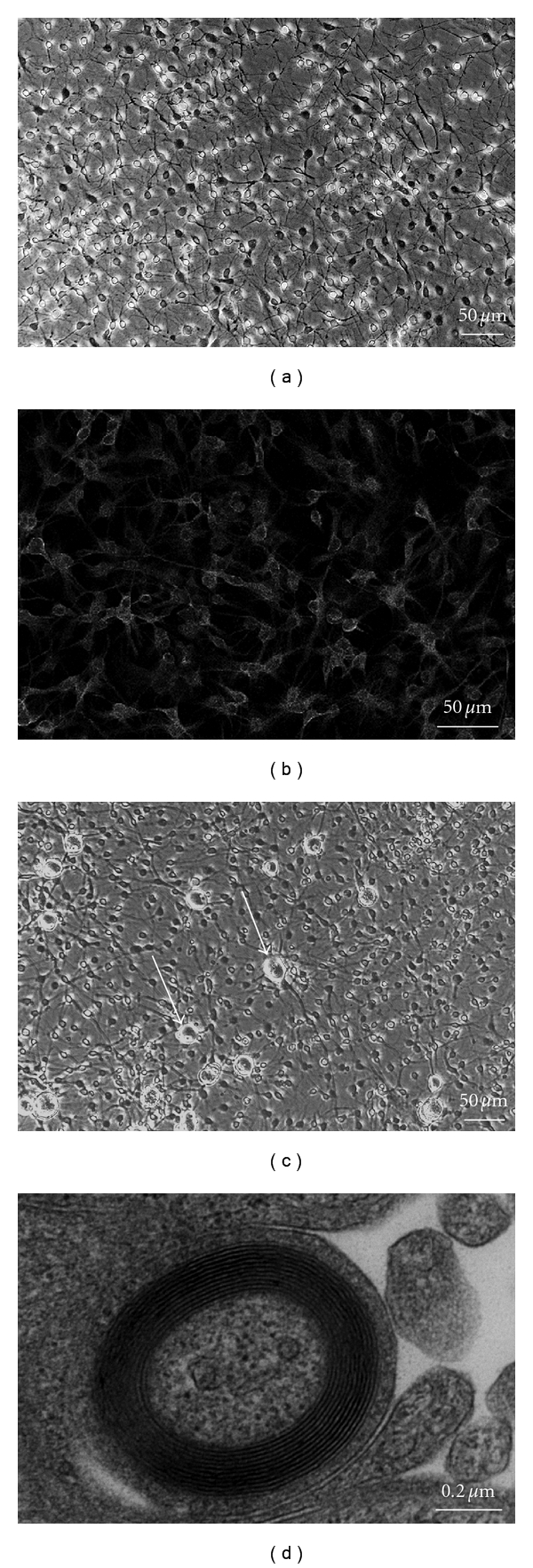
IFRS1 showed distinct Schwann cell phenotypes such as spindle-shaped morphology (a), immunoreactivity to vimentin (b), and myelination in coculture with adult rat DRG neurons (c, d). After 3 days of coculture (c), DRG neurons (arrows) and IFRS1 cells are observed under a phase-contrast microscope. After 28 days of coculture (d), myelin formation is illustrated by electron microscopy.

 We have established immortalized Schwann cells from adult Fischer 344 rats in a similar manner to the mouse cell lines with slight modifications [22]. One of these cell lines, designated IFRS1, showed distinct Schwann cell phenotypes, such as the spindle-shaped morphology (Figure 3(a)) and intense immunoreactivity for S100, p75 ^NTR^, GFAP, laminin (not shown), and vimentin (Figure 3(b)). IFRS1 cells expressed transcription factors (Krox20, Oct6, and SOX10) and myelin proteins (P0, PMP22, and MAG) crucial for Schwann cell development and peripheral myelin formation, in addition to neurotrophic factors (NGF, GDNF, and CNTF), neurotrophin receptors (truncated TrkB, TrkC), and cell adhesion molecules (L1, NCAM, and N-cadherin) required for the survival and neurite outgrowth of neurons and the maintenance of neuron-Schwann cell interactions. In contrast to IMS32 cells, growth stimulants such as neuregulin-*β* and forskolin are needed for the growth and passage of IFRS1 cells. To investigate the ability of IFRS1 cells to myelinate neurites, we cocultured IFRS1 cells with adult rat DRG neurons in the serum-free medium (F12/B27) supplemented with 50 *μ*g/mL ascorbic acid and 10 ng/mL GDNF [72] (Figure 3(c)). In this coculture system, excess proliferation of IFRS1 cells was prevented by the absence of exogenous neuregulin-*β* and forskolin. In addition, neuroprotective molecules secreted from IFRS1 cells helped the cocultured DRG neurons to survive for up to 4 weeks in serum-free conditions. After 28 days of coculture, myelin formation was illustrated by light and electron microscopy (Figure 3(d)). These findings suggest that IFRS1 cells retain the characteristic features of mature Schwann cells and the fundamental ability to myelinate axons, thereby being a valuable tool for exploring neuron-Schwann cell interactions.

**Figure 4:**
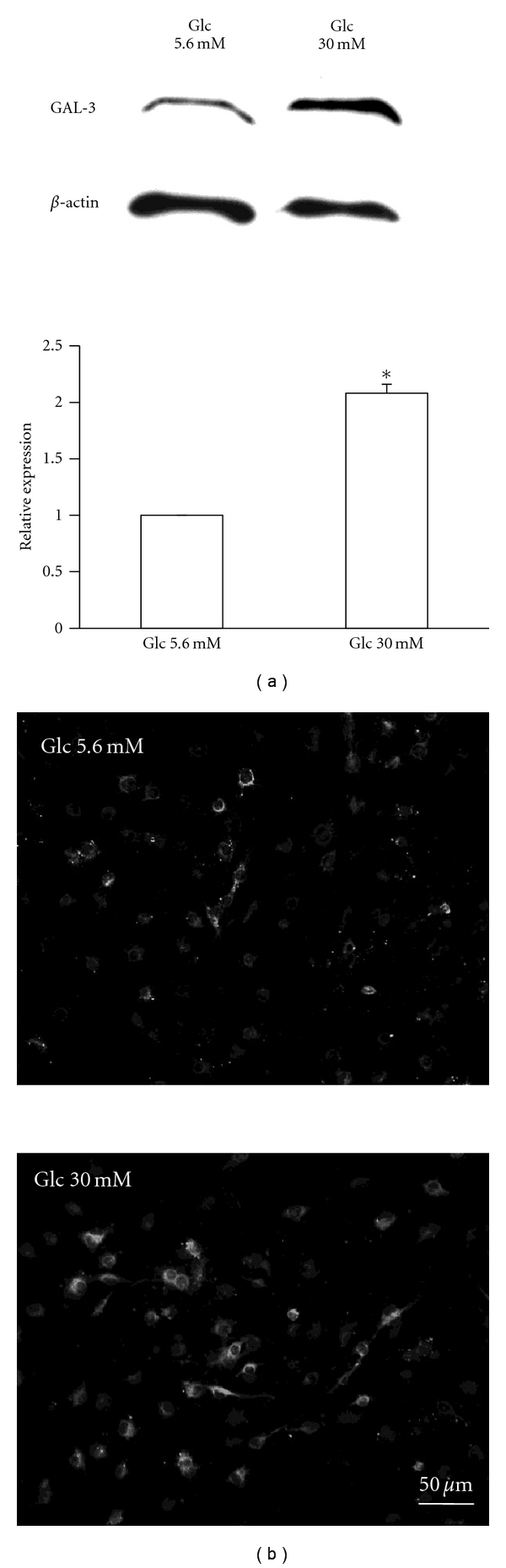
Expression of galectin-3 (GAL-3) in IFRS1 cells after 7 days of exposure to normal (Glc 5.6 mM) and high glucose conditions (Glc 30 mM). (a) Western blot analysis; the representative picture of the blot (upper) and quantitative data (lower) are shown. Values represent the mean + SEM of 3 experiments. *P < .01 as compared with [Glc-5.6 mM]. (b) Immunocytochemical localization of GAL-3 in IFRS1 cells. The photomicrographs show more intense immunoreactivity for GAL-3 in [Glc 30 mM] than that in [Glc-5.6 mM].

 It remains to be elucidated if IFRS1 cells and/or DRG neurons/IFRS1 cell cocultured system under hyperglycemic and/or hyperlipidemic conditions can be a suitable model for the study of diabetic neuropathy. Unlike IMS32 cells, neither AR expression nor intracellular polyol levels were enhanced by exposure of IFRS1 cells to a high glucose (30 mM) condition (Sango et al., unpublished data). However, our preliminary study showed the high glucose-induced upregulation of galectin-3 (GAL-3) in IFRS1 cells (Figure 4). GAL-3 is a member of a family of *β*-galactoside-binding animal lectin and regulates cell-to-cell and cell-to-matrix interactions. Like RAGE, *p60* (AGE-R1), and *p90* (AGE-R2), GAL-3 is identified as an AGE-binding protein [73]. Aragno et al. [74] reported that the upregulation of RAGE and GAL-3 in the hippocampus of STZ-diabetic rats was inhibited by treatment with antioxidants. Also, it is of interest to note that exogenous GAL-3 inhibits proliferation of Schwann cells in cultured sciatic nerve segments [75]. Consistent with this finding, GAL-3 knockout mice show earlier functional recovery and faster regeneration after sciatic nerve crush than the wild-type animals [76]. Further studies are needed to determine whether the upregulation of GAL-3 in Schwann cells under diabetic conditions is involved in the pathogenesis of diabetic neuropathy such as glycation, oxidative stress, and reduced regenerative capability.

## 5. Conclusion

 The spontaneously immortalized Schwann cell lines, such as IMS32 and IFRS1, retain the characteristic features of mature Schwann cells. Considering that an increase in glucose concentration to 20–30 mM accelerated the polyol pathway in IMS32 cells, but not in other Schwann cells, the culture system of IMS32 under high glucose conditions may provide useful mechanistic information about the pathogenesis of diabetic neuropathy, especially polyol pathway-related abnormalities. IFRS1 cells are capable of myelinating neurites in coculture with DRG neurons, and this coculture model can be a valuable tool for exploring neuron-Schwann cell interactions under normal and diabetic conditions.

## Figures and Tables

**Table 1:** Immortalized adult mouse Schwann cell lines established at the authors' institutions.

Line	Origin (murine disease model)	Mouse strain	Ref. no.
MS1	Wild-type	ICR	[19]
IMS32	Wild-type	ICR	[21, 25]
SPMS9	*spm/spm* (Niemann-Pick disease type C)	C57BL/KsJ	[26]
573C10	*npc^nih^/npc^nih^* (Niemann-Pick disease type C)	BALB/c	[25]
TwS1	Twitcher (globoid cell leukodystrophy (Krabbe))	C57/BL6J	[27]
675C20	*P0−/−* (Charcot-Marie-Tooth disease type 1B)	C57/BL6	[25]
654C1	*Nfl^Fcr^/+* (Neurofibromatosis type I)	C57/BL6J	[25]
1113C1	*Hexb−/−* (G_M2_ gangliosidosis (Sandhoff))	C57/BL6	[28]
1089C1	*α-Gal A (−/0)* (Fabry disease)	C57/BL6	[29]

All the Schwann cell lines except MS1 (cells transfected by SV40 large T antigen gene) were established by spontaneous immortalization.
